# A Meta-Analysis of Resveratrol Protects against Myocardial Ischemia/Reperfusion Injury: Evidence from Small Animal Studies and Insight into Molecular Mechanisms

**DOI:** 10.1155/2019/5793867

**Published:** 2019-04-28

**Authors:** Zhi-Jie Mao, Hui Lin, Jian-Wen Hou, Qian Zhou, Qian Wang, Yi-He Chen

**Affiliations:** ^1^Department of Cardiology, The First Affiliated Hospital of Wenzhou Medical University, 325000, Nanbaixiang, Wenzhou, Zhejiang, China; ^2^Department of Respiratory, The Second Affiliated Hospital and Yuying Children's Hospital of Wenzhou Medical University, 325000, Nanbaixiang, Wenzhou, Zhejiang, China; ^3^Department of Cardiology, Xinhua Hospital Affiliated to the Medical School of Shanghai Jiaotong University, 1665 Kongjiang Road, Shanghai 200092, China

## Abstract

**Aims:**

Myocardial ischemia/reperfusion (I/R) injury is a leading cause of cardiomyocyte loss and subsequent ventricular dysfunction after restoring the coronary blood flow and contributes to considerable increase in morbidity and mortality. Resveratrol has been declared to confer cardioprotection against *in vivo* and ex vivo myocardial I/R injury. Here, we have sought to investigate the effects of preconditioning with resveratrol on myocardial I/R damage across the small animal studies.

**Methods and Results:**

The MEDLINE, Google Scholar, PubMed, and Cochrane databases were searched for preclinical studies investigating resveratrol vs. vehicle published from the inception to July 2018. Eventually, 10 *in vivo* and 7 ex vivo studies with 261 animals (130 for resveratrol; 131 for vehicle) were included for meta-analysis. Pooled estimates for primary outcomes demonstrated that pretreatment with resveratrol significantly reduced the infarct size after myocardial I/R injury irrespective of *in vivo* (weighted mean difference (WMD): -13.42, 95% CI: -16.63 to -10.21, *P* ≤ 0.001) or ex vivo (WMD: -15.05, 95% CI: -18.23 to -11.86, *P* ≤ 0.001) studies. Consistently, stratified analysis according to the reperfusion duration, route of administration, or timing regimen of pretreatment all showed the infarct-sparing benefit of resveratrol. Metaregression did not indicate any difference in infarct size based on species, sample size, state, route of administration, reperfusion duration, and timing regimen of pretreatment. Meanwhile, sensitivity analysis also identified the cardioprotection of resveratrol with robust results in spite of significant heterogeneity.

**Conclusions:**

Preconditioning with resveratrol appears to prevent the heart from I/R injury in comparison with vehicle, as evidenced by limited infarct size in a preclinical setting. Studies with large animals or randomized controlled trials will add more evidence and provide the rationale for clinical use.

## 1. Introduction

Acute myocardial infarction is the leading cause of disability and mortality worldwide [1]. Although timely and effective revascularization (i.e., percutaneous coronary intervention, thrombolytic therapy, or coronary artery bypass graft) results in reduction in infarct size, the process of myocardial reperfusion is associated with a further death of cardiomyocytes, which contributes up to 50% of final myocardial damage [2, 3]. So far, the cellular and molecular mechanism underlying myocardial I/R injury remains unclear; experimental evidences show that oxidative stress, inflammation, apoptosis, or calcium overload is deeply involved [2–5]. For decades, novel strategies mitigating lethal reperfusion injury in addition to current reperfusion treatments have been intensively investigated.

Resveratrol is a natural polyphenolic compound, mainly found in edible plants such as peanut, grape, and berry [6]. Moreover, it is also abundant in red wine. Previous studies have demonstrated that resveratrol attenuates the pathological progression in a variety of disease models (i.e., diabetes mellitus, cancer, or neurodegenerative disease) [7–9]. Importantly, it has been currently reported to confer a promising cardioprotective effect against ischemic heart disease *in vivo*, especially myocardial I/R injury, by modulating angiogenesis, oxidative stress, inflammatory, cardiomyocyte apoptosis, and mitochondrial function, along with energy metabolism [10–16]. Furthermore, it also prevents the heart from fibrotic remodeling and hypertrophy. Well-designed experimental studies could provide a deep insight into the efficacy of resveratrol; nevertheless, there are still many discrepancies between the preclinical and the clinical studies due to the complexity of the clinical situation which therefore preclude further application.

Thus, we conduct a comprehensive systematic review and meta-analysis to assess the critical role of resveratrol on myocardial I/R injury across the *in vivo* and ex vivo small animal studies.

## 2. Methods

### 2.1. Search Strategy

We systematically searched the MEDLINE, Google Scholar, PubMed, and Cochrane databases for evidence of the cardioprotective effect of resveratrol in an animal model of myocardial I/R injury published from the inception to July 2018, without any language restriction. The following terms were used for the search: “ischemia/reperfusion injury” or “ischemia-reperfusion injury” or “I/R injury” AND “resveratrol”. In addition, we scrutinized the reference of review articles, meeting abstracts, and comments for additional citations.

### 2.2. Inclusion and Exclusion Criteria

Studies that met the following inclusion criteria were included for further meta-analysis: (1) reported the infarct size determined by a recognized method (i.e., Evans blue/TTC staining for *in vivo* studies or only TTC staining for ex vivo studies). After reperfusion, the coronary artery was reoccluded, and Evans blue was injected intravenously to identify the area at risk. The heart was then excised, sliced, and incubated in TTC to denote the infarct size for *in vivo* studies. And for *ex vivo* studies, only TTC staining was used for evaluating the infarct size after reperfusion; (2) resveratrol vs. vehicle treatment; (3) nonhuman setting; (4) all the procedures and animal care were confirmed to the Guide for the Care and Use of Laboratory Animals published by the United States National Institutes of Health (NIH publication No. 85-23, revised 1996), and the animals were anesthetized before sacrifice, and the heart was excised for further analysis; and (5) no additional anti-inflammatory drugs were used. The exclusion criteria are as follows: (1) studies including animals with cardiovascular comorbidity (i.e., diabetes or obesity), (2) animals treated with resveratrol analogues, and (3) *in vitro* studies.

### 2.3. Data Extraction

Two reviewers (Zhi-Jie Mao and Hui Lin) extracted the data independently from included studies, and discrepancies were resolved by consensus. The following information of each study was extracted and summarized in Table 1: (1) studies' characteristics (i.e., author's name, state, year of publication, number of included animals, and duration of I/R injury), (2) animals' characteristics (i.e., species, sex, body weight/age, and anesthetics), (3) information on interventions (i.e., route of administration, dosage, type of vehicle, and time of treatment), and (4) data about the infarct size of both approaches (to minimize the publication bias, mean and standard deviation rather than standard error were used for further analysis).

### 2.4. Quality Assessment

The quality of included studies was assessed and graded by two reviewers (Yi-He Chen and Hui Lin) based on published criteria for animal experiments [17]. Each of the following was scored as one point: peer-reviewed publication, random allocation to groups, blinded assessment of outcome, sample size calculation, compliance with animal welfare regulations, and a statement of a potential conflict of interest. Discrepancies were resolved by consensus or another reviewer (Jian-Wen Hou) when necessary.

### 2.5. Statistical Analysis

We performed two separate analyses for *in vivo* and ex vivo studies, respectively. Weighted mean difference (WMD) measured the difference of means for infarct size from each included studies and therefore reflects the efficacy of resveratrol treatment. The WMD and respective 95% CIs were measured for continuous variables by using DerSimonian and Laird random effects meta-analysis. The extent of heterogeneity among studies was assessed with Cochran's *Q* test and further quantified by *I*^2^ statistics, which determined the inconsistency across results and presented the proportion of total variation in study estimates that was due to heterogeneity rather than sampling error. Evidence for potential publication bias was evaluated by Begg's and Egger's test. Begg's test assessed if there was a significant correlation between the ranks of the effect estimates and the ranks of their variances. Egger's test used linear regression to assess the relation between the standardized effect estimates and the standard error. Thus, both tests indicated whether the pooled results were affected by publication bias. We stratified the meta-analysis of the primary results by the route of administration (i.p. or i.v.) and timing regimen of pretreatment (short-term or long-term). Sensitivity analysis was conducted by removing one study in turn to estimate the influence of each study. Metaregressions were conducted to explore the impact of potential effect modifiers (species, sample size, state, route of administration, reperfusion duration, and timing regimen of pretreatment) on outcomes and the possible sources of heterogeneity. Statistical analyses were performed with STATA version 12.0 (STATA Corporation, College Station, TX, USA), with *P* values ≤ 0.05 considered statistically significant.

## 3. Results

**Figure 1:**
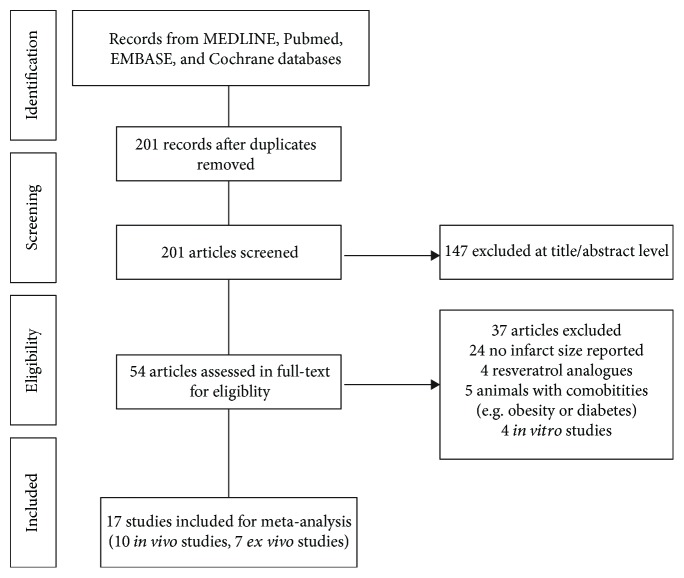
Flow diagram of the study selection process.

Of 201 records identified in the initial search, 147 were removed after title and abstract screening, and the remaining 54 records were retrieved for more detailed evaluation. As a result, 17 literatures (including 10 *in vivo* and 7 ex vivo studies) met our selection criteria (Figure 1). Baseline characteristics of each study were summarized in Table 1 and Table 2. A total of 261 animals were enrolled for comparing resveratrol (*n* = 130) vs. vehicle (*n* = 131) in the setting of myocardial I/R injury. All the eligible studies except one used rodents; however, all the animals included were male. Myocardial I/R injury was achieved by establishing the *in vivo* left coronary artery occlusion-reperfusion model or ex vivo Langendorff-perfused heart model. For *in vivo* studies, animals were preconditioned with resveratrol by either intravenous or intraperitoneal injection. Accordingly, Evans blue/TTC staining was used for assessing the infarct size post-myocardial I/R injury *in vivo*; nonetheless, only TTC staining was performed in *ex vivo* studies, while for *ex vivo* studies, resveratrol was orally administrated or perfused before the assault. The dosing and time regimen of resveratrol treatment varied substantially among the studies. In addition, the majority of these studies were conducted in China (*n* = 9), with the remaining in the USA (*n* = 6), Africa (*n* = 1), or Russia (*n* = 1). The score of included studies ranged from 2 to 4, with a median of 3 out of 6, which may possibly suggest a low risk of bias (Table 3). Furthermore, Table 4 and Table 5 listed the potential molecular and cellular mechanisms of resveratrol in protecting the heart from I/R damage in *in vivo* and ex vivo studies.

### 3.1. *In Vivo* Studies

**Figure 2:**
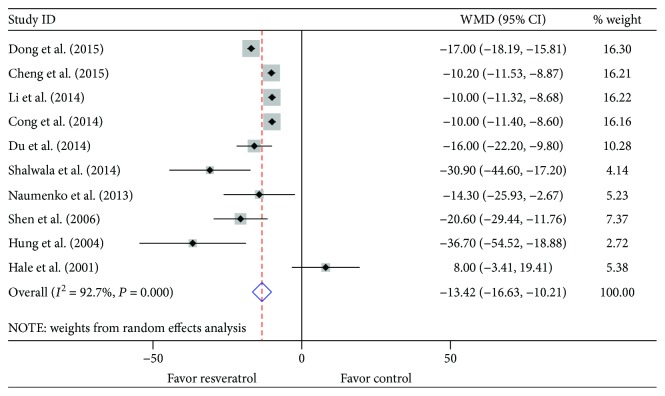
Pooled estimates of infarct size for resveratrol vs. vehicle *in vivo*. Treatment with resveratrol was associated with a smaller infarct size in *in vivo* studies (WMD: -13.42, 95% CI: -16.63 to -10.21, *P* ≤ 0.001). Gray squares represent WMDs in studies. The 95% CIs for each studies are denoted by lines and those for the pooled WMDs by open diamonds. Meta-analysis is performed by random effects model.

**Figure 3:**
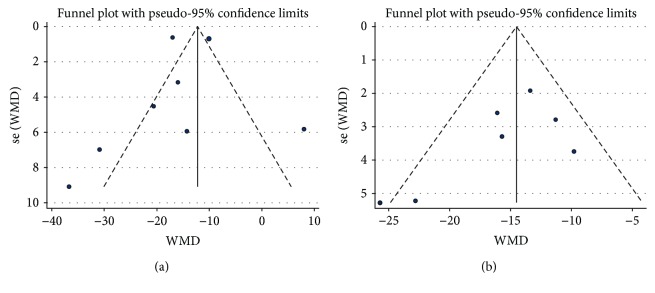
Funnel plot for assessment of publication bias for the infarct size *in vivo* (a) and ex vivo (b). Funnel plots were scatter plots (blue points) of the effect sizes of the included studies versus a measure of their precision usually standard error. The funnel plot appeared to have minimal asymmetry in (a); however, it was symmetrical in (b).

In the pooled analysis using a random effects model, preconditioning with resveratrol in vivo markedly diminished the infarct size when compared with vehicle treatment (WMD: -13.42, 95% CI: -16.63 to -10.21, *P* ≤ 0.001) (Figure 2). There were evidences of high heterogeneity among the studies (*I*^2^ = 92.7%, *P* ≤ 0.001). Absence of publication bias was identified by Begg's (*P* = 0.210) and Egger's test (*P* = 0.673) in spite of a minimal asymmetrical funnel plot (Figure 3(a)). In addition, by systematically excluding each study, the infarct size was still significantly reduced with resveratrol over vehicle treatment in the myocardial I/R injury setting. Notably, stratified analysis suggested that the pooled estimates for improvement of infarct size did not depend on the reperfusion duration, route of administration, or timing regimen of pretreatment (Table 6). Metaregression did not unmask a significant impact of covariates (i.e., species, sample size, state, route of administration, reperfusion duration, and timing regimen of pretreatment) on the beneficial effect of resveratrol (Table 7).

### 3.2. Ex Vivo Studies

**Figure 4:**
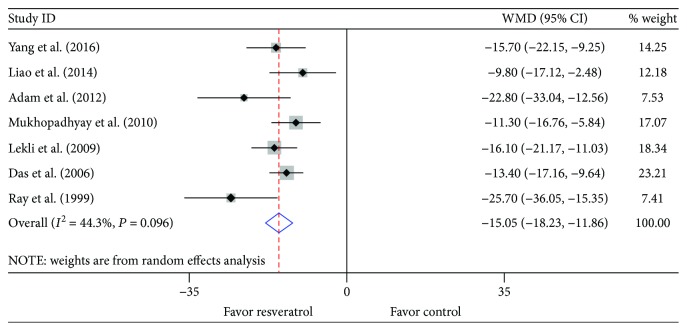
Pooled estimates of infarct size for resveratrol vs. vehicle *ex vivo*. Resveratrol treatment reduced the infarct size in *ex vivo* studies (WMD: -15.05, 95% CI: -18.23 to -11.86, *P* ≤ 0.001). Gray squares represent WMDs in studies. The 95% CIs for each studies are denoted by lines and those for the pooled WMDs by open diamonds. Meta-analysis is performed by the random effects model.

In accordance with the data from *in vivo* studies, administration of resveratrol *ex vivo* was also associated with a significant limitation in infarct size when compared with vehicle treatment (WMD: -15.05, 95% CI: -18.23 to -11.86, *P* ≤ 0.001) (Figure 4). There was evidence of moderate heterogeneity among the studies (*I*^2^ = 44.3%, *P* = 0.096). No publication bias was detected both visually (Figure 3(b)) or mathematically (Begg's test: *P* = 0.368; Egger's test: *P* = 0.155). Unsurprisingly, sensitivity analysis by systematically removing each study provided a consistent estimation of the benefit of resveratrol treatment in reducing the infarct size. Stratified analysis by reperfusion duration, route of administration, or timing regimen of pretreatment had no impact on the effect size and the *P* value (Table 6). Similarly, there was no relationship between the prespecified covariates and pooled estimates by metaregression (Table 7).

## 4. Discussion

To our knowledge, this is the first preclinical meta-analysis to investigate the cardioprotective effect of resveratrol in animals subjected to myocardial I/R injury. Our findings indicate that as compared with vehicle, resveratrol is associated with a significantly improved infarct size of hearts post-I/R injury in both *in vivo* and ex vivo small animal studies. The marked benefits of resveratrol are not affected by either the duration of reperfusion or route and timing regimen of administration.

Reperfusion injury is a devastating consequences for reestablishment of blood flow to the ischemic myocardium, which induced additional damage inflicted on the heart [2]. It is first reported by Jennings and Reimer that reperfusion exacerbated the cell necrosis of irreversible injured cardiomyocytes [18]. Subsequently, experimental and clinical studies also confirm the paradoxical phenomenon and further investigate the cellular and molecular mechanism underlying the pathophysiological progress [1–3]. Although not fully elucidated, accumulating evidence has demonstrated a causality between the myocardial I/R injury and intracellular calcium overload, inflammation, and oxidative stress [4, 5, 19]. Mitochondrial dysfunction is recognized as the main source of reactive oxygen species in the pathogenesis of reperfusion injury and also promotes inflammatory response and endothelial damage [20]. Meanwhile, mPTP opening and subsequent cytochrome c released from impaired mitochondria trigger the intrinsic apoptotic process by activation of caspase-9/3 signaling pathway. Cardiomyocyte apoptosis along with necrosis collectively contributes to an extended infarct size post-I/R injury. Autophagy has also played an important role in the development of reperfusion damage; previous researches show that activation or inhibition of autophagy could exert either a beneficial or detrimental effect in the context of myocardial I/R injury [21]. In addition, platelet aggregation induced by I/R injury contributed to microvascular obstruction, characterized by microcirculatory spasm, intraluminal thrombosis, and notably swollen and dysfunctional endothelial cells, and finally caused slow or no-reflow [22]. Based on the aforementioned results, it provides the rationale for therapeutic strategies targeted against these adverse pathways.

Resveratrol is a unique plant-derived polyphenol and has been demonstrated to exhibit impressively beneficial effects in attenuating the progression of various illness, including ageing, obesity, cancer, inflammatory bowel disease, depressant, and diabetes mellitus, along with cardiovascular disease [6, 23]. The biological and pharmacological properties of resveratrol have been well established, i.e., antioxidant, anti-inflammation, antimitochondrial dysfunction, and antiapoptotic potency. Moreover, metabolic modulation and angiogenesis are also identified as the therapeutic actions of resveratrol [24]. It is worth noticing that resveratrol exerted cardioprotection on ischemic heart disease, especially the myocardial I/R injury. After reperfusion injury, inflammatory response and oxidative stress mainly contribute to the substantial loss of myocytes and consequent enlarged infarct area. An experimental study by Dong et al. shows that resveratrol protects the myocardium against I/R damage through deactivation of NALP3 inflammasome and suppression of IL-1*β*- and IL-18-mediated inflammatory cascade [10]. Neutrophils are also deeply implicated in the inflammatory response, and robust accumulation of neutrophils in reperfused areas results in negative repercussions for cardiomyocyte survival [25]. In addition, impaired mitochondria and infiltrated immune cells cause substantial ROS generation and consequent excessive oxidative stress [20]. Evidence from previous studies have identified resveratrol as an antioxidant that regulates the multistep process of redox system [19]. Recently, pretreatment with resveratrol decreases the ROS level by DCFH-DA staining, inhibits MDA formation, and is inversely correlated with increased expression of antioxidant enzymes, i.e., MnSOD and catalase in both *in vivo* and ex vivo myocardial I/R models [11, 14]. Meanwhile, inflammation and/or oxidative stress evidently trigger apoptosis cascades via either intrinsic or extrinsic apoptotic signaling pathway leading to cardiomyocyte loss and adverse ventricular remodeling, further deteriorating contractile function post-myocardial I/R injury. In accordance with the favorable effects of resveratrol in scavenging ROS production and alleviating inflammatory response, apoptosis assessed by TUNEL staining is also markedly diminished by resveratrol administration in the heart of I/R damage [12]. Similarly, resveratrol prevents mPTP opening, cytochrome c release from mitochondria, and subsequent caspase-3 activation during I/R injury and thus protects against mitochondrial dysfunction-induced cell death [15]. Noteworthy, contemporary studies find that resveratrol conferred vasoprotection through attenuating endothelial dysfunction and prompt angiogenesis in the reperfused myocardium, evidenced by restored expression of eNOS, nNOS, and VEGF-B [14, 26]. Intriguingly, pretreatment with a NOS inhibitor (L-NAME) or cGMP inhibitor (MB) significantly abolished the cardioprotection of resveratrol, which demonstrated a critical role of proangiogenic effect underlying the impressively beneficial effects of resveratrol [12]. Importantly, it has been reported that TLR4/NF-*κ*B signaling pathway is involved in the biological effects of resveratrol [12]. TLR4 is rapidly upregulated to mediate a multitude of proinflammatory cytokines in response to I/R injury, which conspire to induce myocardial damage [27]. Subsequently, it predominantly activates the NF-*κ*B family, the key transcription factors in modulating the inflammatory response, and cell death genes linked to the pathogenesis of cardiovascular disease. Surprisingly, resveratrol treatment decreases the expression of TLR4 and NF-*κ*B in reperfused myocardium, accompanied with lower levels of TNF-*α* and reduced infarct size. However, whether there is a causal relationship between resveratrol and TLR4/NF-*κ*B signaling needed further investigation. Other studies suggest Nrf2, SIRT1, and AKT/GSK3*β* as potential targets of resveratrol [11, 14, 16, 28]. Nevertheless, of course, detailed molecular and cellular mechanisms of resveratrol are far more sophisticated than we have got from the experimental results (Table 4 and Table 5). Unexpectedly, there is very little success in transforming resveratrol into clinical practice with a desired efficacy in relevant patients in spite of promising infarct-limiting effects from animal studies. Theoretically, animal models help to explore the probable mechanism; however, there is still a huge anatomic and/or physiological gap between the different species which may possibly be responsible for the inconsistency between preclinical studies and clinical studies. Since the overall conclusion of this work mainly depends on evidence from animal studies, large animal studies and/or well-designed RCTs are of pressing need for evaluating the expected cardioprotection of resveratrol.

## 5. Limitation

Several limitations should be considered: First, we performed meta-analysis at an aggregate study level (based on mean value and standard deviation) due to unavailability of individual animal-level data. We could not conduct stratified analyses to evaluate the impact of treatment in relation to other relevant variables (i.e., body weight, age, or level of left ventricular dysfunction). Second, our results can only be generalized to the overall animals subjected to myocardial I/R injury without any cardiovascular comorbidities (i.e., diabetes mellitus, obesity, hypertension, hyperlipidemia, or even renal dysfunction). Third, it was worthwhile to note that the absence of data on large animals in our statistical analysis, which shared more pathophysiological characteristics with human, may also possibly limit the interpretation and extension of our results and thus warranted large animal studies to further confirm the favorable effects of resveratrol on the heart with I/R damage. Fourth, variation in route of pretreatment, dosing, and timing regimens may have a possible impact on the cardioprotection of resveratrol. Finally, we found statistical heterogeneity among the studies; however, the robustness of the data across sensitivity analysis and stratified analysis can help minimize the potential effect of heterogeneity on the reliability of our conclusions. Furthermore, metaregression also did not reveal any possible source which may be responsible for the high heterogeneity due to the consistent effect of resveratrol irrespective of study level covariates.

## 6. Conclusion

From the available data of small animal studies, preconditioning with resveratrol presented a favorable infarct-limiting effect against myocardial I/R damage. Nonetheless, large animal studies and well-designed randomized controlled trials were needed in order to further confirm the cardioprotection of resveratrol before clinical application.

## Figures and Tables

**Table 1:** Characteristics of the *in vivo* studies included in the meta-analysis.

Author	Year	State	Species	Weight/year	Anesthetic	Animal numbers		I/R duration	Vehicle	Resveratrol treatment		
						Control	Resveratrol			Dosage	Approach	Time
Dong et al. [10]	2015	China	Rats, SD, M	270-300 g	Pentobarbital	10	10	30 min/2 h	DMSO	10 mg/L	i.p.	60 min before I/R
Cheng et al. [11]	2015	China	Rats, SD, M	250-300 g	Sodium pentobarbital	10	10	30 min/2 h	0.9% NaCl	100 *μ*mol/L	i.v.	5 min before I/R
Li et al. [12]	2014	China	Rats, SD, M	250-300 g	Sodium pentobarbital	10	10	30 min/2 h	0.9% NaCl	100 *μ*mol/L	i.v.	5 min before I/R
Cong et al. [13]	2014	China	Rats, SD, M	250-300 g	Sodium pentobarbital	10	10	30 min/2 h	0.9% NaCl	100 *μ*mol/L	i.v.	5 min before I/R
Du et al. [29]	2014	China	Mice, M	3 months	2% isoflurane	8	8	30 min/24 h	Ethanol	10 mg/L	i.p.	60 min before I/R
Shalwala et al. [28]	2014	USA	Mice, ICR, M	35.5 ± 5 g	Sodium pentobarbital	6	6	30 min/24 h	DMSO	5 mg/L	i.p.	24 h before I/R
Naumenko et al. [30]	2013	Russia	Rats, Wistar, M	339.5 ± 9.8 g	Urethane	10	9	30 min/2 h	DMSO	10 *μ*mol/L	i.v.	30 min before I/R
Shen et al. [31]	2006	China	Rats, SD, M	275-300 g	Sodium pentobarbital	8	8	30 min/2 h	DMSO	10 *μ*mol/L	i.v.	15 min before I/R
Hung et al. [26]	2004	China	Rats, SD, M	270-300 g	Urethane	8	8	60 min/3 h	DMSO	1 mg/L	i.p.	60 min before I/R
Hale and Kloner [32]	2001	USA	Rabbits, NZW, M	2.2-3.0 kg	Ketamine/xylazine	8	8	30 min/3 h	Ethanol	1.5 mg/L	i.v.	15 min before I/R

SD: Sprague-Dawley rats; ICR: Institute of Cancer Research mice; NZW: New Zealand White rabbit; M: male; DMSO: dimethyl sulfoxide; i.p.: intraperitoneal injection; i.v.: intravenous injection.

**Table 2:** Characteristics of the ex vivo studies included in the meta-analysis.

Author	Year	State	Species	Weight/year	Anesthetic	Animal numbers		I/R duration	Vehicle	Resveratrol treatment		
						Control	Resveratrol			Dosage	Approach	Time
Yang et al. [14]	2016	China	Rats, SD, M	10 weeks	Chloral hydrate	10	10	30 min/1 h	K-H solution	10 *μ*mol/L	Perfusion	15 min before I/R
Liao et al. [15]	2014	China	Mice, KM, M	NA	Sodium pentobarbital	6	6	15 min/30 min	Ethanol	2.0 mg/kg.d	Gavage	6 weeks before I/R
Adam et al. [16]	2012	Africa	Rats, LE, M	9 weeks	Sodium pentobarbital	6	6	30 min/1 h	K-H solution	10 mmol/L	Perfusion	25 min before I/R
Mukhopadhyay et al. [33]	2010	USA	Rats, SD, M	250-300 g	Sodium pentobarbital	6	6	30 min/2 h	NA	5 mg/kg/d	Gavage	21 days before I/R
Lekli et al. [34]	2009	USA	Rats, SD, M	250-300 g	Sodium pentobarbital	3	3	30 min/2 h	NA	2.5 mg/kg/d	Gavage	15 days before I/R
Das et al. [35]	2006	USA	Rats, SD, M	250-300 g	Sodium pentobarbital	6	6	30 min/2 h	K-H solution	10 *μ*mol/L	Perfusion	20 min before I/R
Ray et al. [36]	1999	USA	Rats, SD, M	275–300 g	Sodium pentobarbital	3	3	30 min/2 h	K-H solution	10 *μ*mol/L	Perfusion	15 min before I/R

SD: Sprague-Dawley rats; KM: Kun-Ming; M: male; LE: Long-Evans; K-H solution: Krebs-Henseleit solution; DMSO: dimethyl sulfoxide.

**Table 3:** The research quality of included studies.

Authors	A	B	C	D	E	F	Score
*In vivo* studies							
Dong et al.	Y	Y	N	N	Y	Y	4
Cheng et al.	Y	Y	N	N	Y	Y	4
Li et al.	Y	Y	N	N	Y	N	3
Cong et al.	Y	Y	N	N	Y	N	3
Du et al.	Y	N	N	N	Y	Y	3
Shalwala et al.	Y	N	Y	N	Y	N	3
Naumenko et al.	Y	Y	N	N	Y	Y	4
Shen et al.	Y	Y	N	N	N	N	2
Hung et al.	Y	N	N	N	Y	N	2
Hale and Kloner	Y	Y	N	N	Y	N	3

*Ex vivo* studies							
Yang et al.	Y	Y	N	N	Y	N	3
Liao et al.	Y	Y	N	N	Y	Y	4
Adam et al.	Y	N	N	N	Y	Y	3
Mukhopadhyay et al.	Y	N	N	N	Y	Y	3
Lekli et al.	Y	Y	N	N	Y	N	3
Das et al.	Y	Y	N	N	Y	N	3
Ray et al.	Y	Y	N	N	Y	N	3

A: peer-reviewed publication; B: random allocation to groups; C: blinded assessment of outcomes; D: sample size calculation; E: compliance with animal welfare regulations; F: a statement of a potential conflict of interest. Y: yes; N: no.

**Table 4:** The proposed molecular and cellular mechanism of the cardioprotective effect of resveratrol in *in vivo* studies.

Studies	Year	Dosage	Proposed mechanism
Dong et al. [10]	2015	10 mg/L	Downregulation of inflammatory response (NALP3 inflammasome, IL-1*β*, IL-18) and caspase-1 expression
Cheng et al. [11]	2015	100 *μ*mol/L	Attenuate inflammation (MPO), oxidative stress (SOD, MDA, GSH-PX) possibly via Nrf2/ARE pathway
Li et al. [12]	2014	100 *μ*mol/L	Deactivation of TLR4/NF-*κ*B signaling, anti-inflammation (MPO, TNF-*α*, NO)
Cong et al. [13]	2014	100 *μ*mol/L	Activation of cGMP/NO signaling, anti-inflammation (MPO, TNF-*α*)
Du et al. [29]	2014	10 mg/L	Activation of AMPK/Kir6.2-containing K-ATP channel signaling
Shalwala et al. [28]	2014	5 mg/L	Activation of SIRT1 signaling
Naumenko et al. [30]	2013	10 *μ*mol/L	NA
Shen et al. [31]	2006	10 *μ*mol/L	Attenuate oxidative stress (MDA, NO)
Hung et al. [26]	2004	1 mg/L	NO-independent
Hale and Kloner [32]	2001	1.5 mg/L	NA

**Table 5:** The proposed molecular and cellular mechanism of cardioprotective effect of resveratrol in ex vivo studies.

Studies	Year	Dosage	Proposed mechanism
Yang et al. [14]	2016	10 *μ*mol/L	Upregulation of VEGF-B signaling, attenuate oxidative stress (ROS, MnSOD)
Liao et al. [15]	2014	2.0 mg/kg d	Upregulation of VDAC1 signaling, inhibit mitochondria-mediated apoptosis (mPTP, caspase-3, cytochrome c)
Adam et al. [16]	2012	10 mmol/L	SIRT1-independent
Mukhopadhyay et al. [33]	2010	5 mg/kg/d	Regulation of miRNA expression (miR-21)
Lekli et al. [34]	2009	2.5 mg/kg/d	Regulation of autophagy
Das et al. [35]	2006	10 *μ*mol/L	Activation of MAPK (ERK 1/2, p38 MAPK)/MSK1/CREB signaling, antiapoptosis
Ray et al. [36]	1999	10 *μ*mol/L	Attenuate oxidative stress (MDA)

**Table 6:** Stratified analysis of pooled estimates of infarct size *in vivo* and *ex vivo*.

Pooled estimates	No. of studies	WMD (95% CI)	*P* value
		*In vivo* studies	
Reperfusion duration			
<24 h	8	-12.26 (-15.67, -8.84)	≤0.001
≥24 h	2	-22.15 (-36.52, -7.77)	0.003
Route of administration			
i.p.	4	-20.57 (-26.81, -14.33)	≤0.001
i.v.	6	-10.14 (-11.98, -8.29)	≤0.001
Timing regimen of pretreatment			
≥60 min	4	-20.57 (-26.81, -14.33)	≤0.001
<60 min	6	-10.14 (-11.98, -8.29)	≤0.001
Overall	10	-13.42 (-16.63, -10.21)	≤0.001

		*Ex vivo* studies	
Reperfusion duration			
≤1 h	3	-15.38 (-21.93, -8.82)	≤0.001
>1 h	4	-15.06 (-19.15, -10.96)	≤0.001
Route of administration			
Perfusion	4	-17.75 (-23.11, -12.40)	≤0.001
Gavage	3	-12.92 (-16.70, -9.14)	≤0.001
Timing regimen of pretreatment			
≥60 min	3	-12.92 (-16.70, -9.14)	≤0.001
<60 min	4	-17.75 (-23.11, -12.40)	≤0.001
Overall	7	-15.05 (-18.23, -11.86)	≤0.001

Stratified analysis investigated whether particular categorical covariates explain any of the heterogeneity of treatments between studies.

**Table 7:** Metaregression analysis *in vivo* and ex vivo.

Covariates	Infarct size (*in vivo* studies)			Infarct size (*ex vivo* studies)		
	Coefficient	95% CI	*P* value	Coefficient	95% CI	*P* value
Species	6.122819	-6.043731; 18.28937	0.279	5.763822	-8.045439; 19.57308	0.332
Sample size	1.662638	-1.379227; 4.704503	0.243	0.2914345	-0.869869; 1.452738	0.547
State	1.953709	-11.39187; 15.29928	0.744	-0.774244	-6.697155; 5.148667	0.750
Route of administration	4.398078	-12.15415; 20.9503	0.557	4.566912	-4.397479; 13.5313	0.247
Reperfusion duration	-9.196691	-29.21135; 10.81797	0.320	1.047275	-5.011723; 7.106273	0.675
Timing regimen of pretreatment	2.226924	-3.267802; 7.721649	0.377	0.7420125	-1.891014; 3.375039	0.501

Metaregression provided valuable information regarding the interaction between the continuous covariates and treatment effect of resveratrol in reducing the infarct size and may explore the source of heterogeneity. Consistent *P* value showed that none of the covariates below had an impact on the cardioprotection of resveratrol in myocardial I/R injury.

## Data Availability

The data used to support the findings of this study are available from the corresponding author upon request.
